# Carbonic anhydrase, its inhibitors and vascular function

**DOI:** 10.3389/fmolb.2024.1338528

**Published:** 2024-01-29

**Authors:** Andrea García-Llorca, Fabrizio Carta, Claudiu T. Supuran, Thor Eysteinsson

**Affiliations:** ^1^ Department of Physiology, Faculty of Medicine, University of Iceland, Reykjavik, Iceland; ^2^ NEUROFARBA Department, Section of Pharmaceutical and Nutraceutical Sciences, University of Florence, Florence, Italy; ^3^ Department of Ophthalmology, Faculty of Medicine, University of Iceland, Reykjavik, Iceland

**Keywords:** carbonic anhydrase, vasculature, blood flow, vascular tone, enzyme isoforms, inhibitors

## Abstract

It has been known for some time that Carbonic Anhydrase (CA, EC 4.2.1.1) plays a complex role in vascular function, and in the regulation of vascular tone. Clinically employed CA inhibitors (CAIs) are used primarily to lower intraocular pressure in glaucoma, and also to affect retinal blood flow and oxygen saturation. CAIs have been shown to dilate vessels and increase blood flow in both the cerebral and ocular vasculature. Similar effects of CAIs on vascular function have been observed in the liver, brain and kidney, while vessels in abdominal muscle and the stomach are unaffected. Most of the studies on the vascular effects of CAIs have been focused on the cerebral and ocular vasculatures, and in particular the retinal vasculature, where vasodilation of its vessels, after intravenous infusion of sulfonamide-based CAIs can be easily observed and measured from the fundus of the eye. The mechanism by which CAIs exert their effects on the vasculature is still unclear, but the classic sulfonamide-based inhibitors have been found to directly dilate isolated vessel segments when applied to the extracellular fluid. Modification of the structure of CAI compounds affects their efficacy and potency as vasodilators. CAIs of the coumarin type, which generally are less effective in inhibiting the catalytically dominant isoform hCA II and unable to accept NO, have comparable vasodilatory effects as the primary sulfonamides on pre-contracted retinal arteriolar vessel segments, providing insights into which CA isoforms are involved. Alterations of the lipophilicity of CAI compounds affect their potency as vasodilators, and CAIs that are membrane impermeant do not act as vasodilators of isolated vessel segments. Experiments with CAIs, that shed light on the role of CA in the regulation of vascular tone of vessels, will be discussed in this review. The role of CA in vascular function will be discussed, with specific emphasis on findings with the effects of CA inhibitors (CAI).

## Introduction

A common metalloprotein found in both prokaryotes and eukaryotes, Carbonic Anhydrase (CAs, EC 4.2.1.1) enzymes catalyze the basic biochemical reaction of carbon dioxide hydration, which results in the production of a bicarbonate anion and a proton. Consequently, they are one of the key regulators of cellular pH homeostasis (Supuran, 2016b). These enzymes also participate in several crucial biological processes, including respiration, the transport of bicarbonate and carbon dioxide ions, the secretion of electrolytes, production of urea, bone resorption, lipogenesis, and gluconeogenesis, and more (Khalifah, 1973; Ekinci et al., 2012; Şentürk et al., 2012). In fact, eight genetically distinct CA families (α, β, γ, δ, ζ, η, θ and ι) are currently recognized. These families contain a variety of metal ions at their active sites, including Zn(II) (in most classes), Fe(II) (specific in the γ), Co(II) (in the δ), and Cd(II) (in the ζ) (Guzel-Akdemir et al., 2013; Maresca et al., 2013; Vullo et al., 2014a; 2014b; Capasso and Supuran, 2014; Del Prete et al., 2014). Whereas the ι-CAs do not have metal ion in their active sites (Hirakawa et al., 2021). Until now 16 α-CA isozymes have been discovered in mammals, each with unique tissue distribution, subcellular localization, and catalytic activity (Guzel-Akdemir et al., 2013; Supuran, 2016b). Two of these isozymes are mitochondrial (CA VA and CA VB), one is secreted in both milk and saliva (CA VI), some of them are cytosolic (CA I, CA II, CA III, CA VII, and CA XIII), while others are membrane-bound (CA IV, CA IX, CA XII, and CA XIV) (Maren, 1967; Alterio et al., 2012; Ekinci et al., 2012; Supuran, 2012; Supuran, 2016b). It has been known for decades that this enzyme family has the potential to be a significant class of biological targets for pharmacological intervention (Aggarwal et al., 2013). Several human diseases, including glaucoma (hCAs I, II, IV, and XII), cancer (hCAs IX and XII), some central-nervous system syndromes, such as epilepsy, neuropathic pain, and idiopathic intracranial hypertension (hCAs I, II, and VII), edema (hCA II, IV, XII, and XIV), obesity (hCA VA and VB), and osteoporosis (hCA IV and XIV), have been associated with an abnormal or dysregulated expression level in the forementioned human diseases (Alterio et al., 2012; De Simone et al., 2013; Carta et al., 2014; Supuran, 2016a).

**FIGURE 1:**
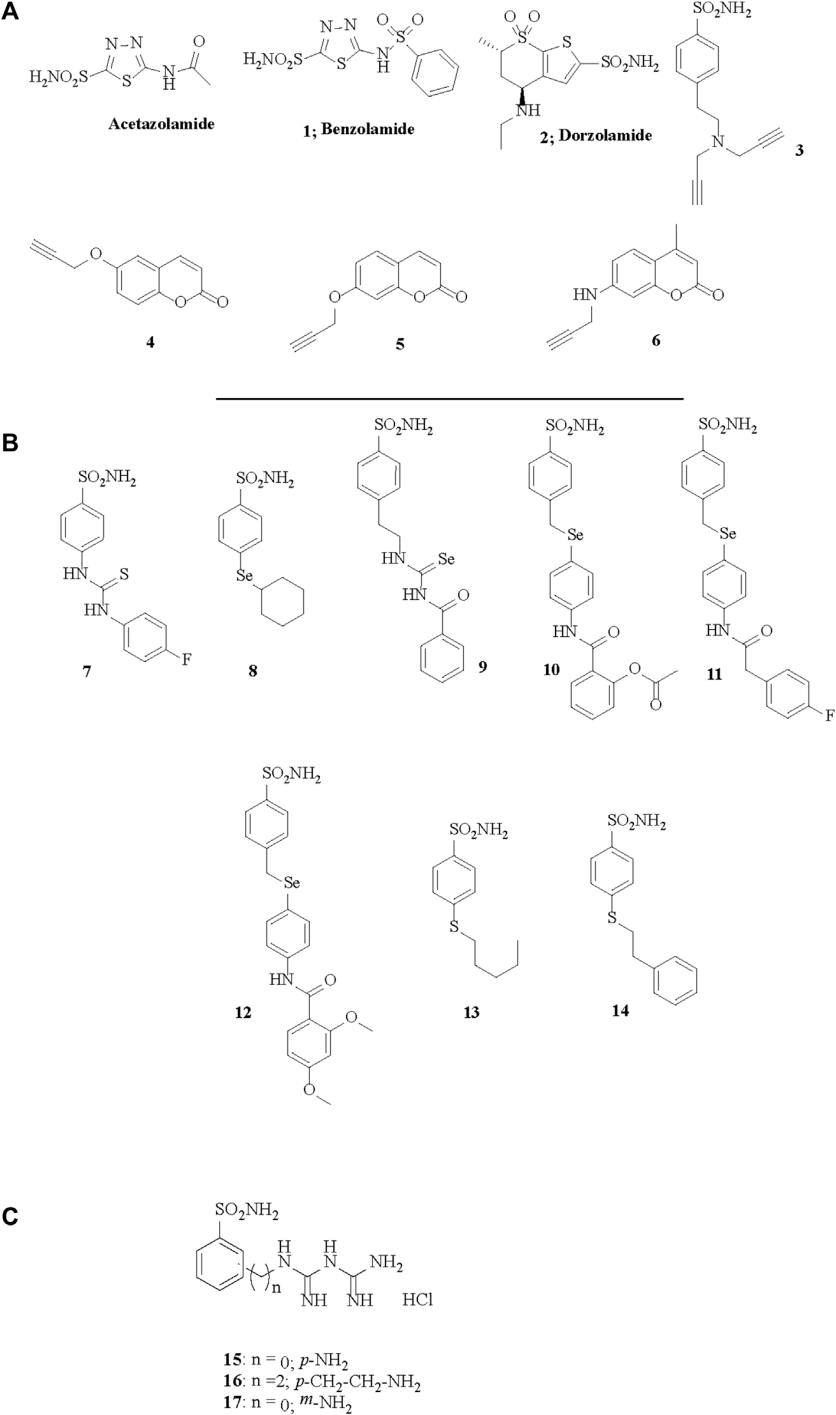
Chemical structures of acetazolamide, the best known sulfonamide inhibitor and of the CAIs **1–17** investigated in **(A)** Eysteinsson et al., 2019, **(B)** Eysteinsson et al., 2022 and **(C)** Eysteinsson et al., 2023.

To date, there are two main categories of CA inhibitors (CAIs): those that directly interact with the metal ion in the active site and those that do not (Alterio et al., 2012). Since their discovery in 1940, primary sulfonamides are the most important and historically significant class of CAIs (Winum et al., 2006), and there have been numerous members of the class in clinical use for decades (Supuran, 2008a). The sulfonamide function, referred to as a zinc-binding group (ZBG), forms hydrogen bonds with adjacent residues such as Thr199 in α-CAs, also coordinating to the Zn (II) ion in the hCA active sites in deprotonated, sulfonamidate form (Supuran, 2008a; 2017; Margheri et al., 2016; Bua et al., 2017). All 16 human isozymes that belong to the α class share these binding properties in their active site designs (Alterio et al., 2012; Carta et al., 2014; Supuran, 2016a). Furthermore, the bioisosteres of sulfonamides (such as sulfamates and sulfamides) demonstrate CA inhibitory activity via a similar mechanism, which is also valid for mercaptophenols, ureates/hydroxamates, metal complexing anion inhibitors, and other less investigated classes of compounds (Alterio et al., 2012; D'Ambrosio et al., 2015; Maresca et al., 2010; Maresca et al., 2009; Maresca et al., 2013). Examples of CAIs structures such as, acetazolamide (Figure 1), methazolamide, (examples of antiglaucoma drugs), topiramate (anticonvulsant therapeutic agent), among others that have been used extensively in the clinic (Ramya et al., 2017). Given that many of the CA isoforms are very similar from a structural perspective and even in terms of subcellular localization, the main disadvantage of using CAIs is their lack of selectivity in inhibiting different isoforms, which leads to unintended side effects (Supuran, 2008a; 2017; Margheri et al., 2016; Bua et al., 2017). Designing selective/specific drugs with discrete inhibitory profiles (inhibitors or activators) for any of these isoforms is therefore still difficult. The ring and tail approaches have been used extensively in recent years in numerous attempts to create isoform-selective sulfonamide inhibitors (Lopez et al., 2010; Alterio et al., 2012; Supuran, 2016a; Supuran, 2023), whereas approaches based on monoclonal antibodies (mAbs) or antibody-drug conjugates are also under consideration (Testa et al., 2022). In recent years it has become increasingly clear that CA and its inhibitors have complex effects on vascular function and regulation of blood flow, including at the level of the vascular wall and vascular cells themselves. In this review, the aim is to shed light on what is known about these effects, and what may be the underlying mechanisms involved.

The function of the vasculature of the body is under a complex set of control mechanisms, some of which involve the nervous system and/or local factors. It must be able to respond to homeostatic changes in both a quick and slow manner, to maintain blood pressure and stable supply of oxygen and nutrients. Between the organs of the body there is a certain degree of anatomical heterogeneity of the blood vessels in each. The structure and function of the blood vessels thus varies to some extent between organs, and the processes involved in the regulation and control of each vascular bed are to a great degree different. The vascular cells involved are the primary basis of these differences, in particular the endothelial cells (ECs), pericytes and vascular smooth muscle cells (Potente and Makinen, 2017). The endothelium in most organs establishes a barrier between the blood and the tissue which is dynamic. Certain commonalities are present in most capillaries: they have a lumen that is about 10 µm in diameter and which is lined with a single layer of ECs. The capillaries of the body have however differences in the morphology and function of their ECs, depending on the organ involved (Augustin and Koh, 2017), and there are major types known: continuous, fenestrated, and sinusoidal. The differences in the morphology and function of these three types of ECs play an important role in determining the heterogeneity of capillaries. Fenestrated capillaries are found in filtration sites in the kidneys, the choroid plexus of the brain, and in several endocrine organs (Augustin and Koh, 2017), i.e., organs that are involved in filtration and secretion (Aird, 2012). Organs with passive and active transport of fluid and larger solutes thus tend to have fenestrated capillaries. Vessels with sinusoids are located in the liver, spleen, bone marrow and endocrine organs such as the adrenal medulla and the pituitary gland (Augustin and Koh, 2017). Sinusoidal ECs in the liver are discontinuous, with an incompletely organized basement membrane and with large fenestrae that are organized in sieve plates (Aird, 2012; Potente and Makinen, 2017). In addition to the structural differences amongst ECs there is heterogeneity of function. There are differences between vascular beds in their capacity to deliver nutrients to the tissue, and in their permeability (Potente and Makinen, 2017). It is likely that these differences in EC morphology and function, and interactions with other vascular cells in the vascular wall of the vessels in question relate to the effectiveness of CA inhibitors as vasodilators in different vascular beds. Still, although CA II and CA IV have been shown to be expressed in the corneal endothelium (Nguyen and Bonanno, 2011), only the membrane bound CA IV and CA IX isoforms have been shown to be present in normal vascular endothelial cells, and not the CA II (Lee et al., 2018; Annan et al., 2019). However, CA II present in other vascular cells may have indirect effects on vascular endothelial function, but the structural and functional heterogeneity of ECs is likely to determine to what extent and by which mechanism CA is involved in regulation of vascular function in the organ concerned.

## Functions of the enzyme carbonic anhydrase

Carbonic anhydrases are eight genetically separate families of enzymes (α to ι-CAs) whose primary function is to catalyze the reversible reaction between carbon dioxide (CO_2_) and bicarbonate (HCO_3_^−^). They are distributed to a variable extent throughout the bodies of all living organisms, but in vertebrates only the α family is present. Within the α family there are 16 different isoforms of the enzyme found, but their distribution in tissue varies. The α family is the best characterized one of all the families of isoforms. In mammalian tissue the α family of CA affects a variety of physiological functions, such as the concentration of CO_2_, HCO_3_^−^, and H^+^ in both extracellular and intracellular compartments, and transport of these across cell membranes, the acid-base balance in the tissue and regulation of intra- and extracellular pH, gas exchange at the air-water interface, vascular calcification, secretion of cytokines, oncogenesis to name some (Supuran, 2016b). For each of these functions specific isoforms of CA are involved or play a major role. The transport function of several acid-base transport cell membrane proteins in mammalian tissue is regulated by carbonic anhydrase. These include the SLC4 family of HCO_3_^−^ transporters, with three distinct Cl^−^/HCO_3_^−^ exchangers (anion exchangers AE1-3), and five Na^+^/HCO_3_^−^ co-transporters (NBCe1, NBCe2, NBCn1, NDCBE and NCBE); the SLC26 family of Cl^−^/HCO_3_^−^ exchangers with six members (SLC26A2, SLC26A3 (DRA), SLC26A4(Pendrin), SLC26A6(PAT-1), SLC26A7, and SLC26A9) and the SLC9 family of Na^+^/H^+^ exchanger membrane proteins (Becker and Deitmer, 2007; Becker et al., 2014). The CA enzyme needs to bind to specific binding sites on these membrane transporter proteins to activate or increase transport, and the catalytic activity of CA is required as well for the transport to occur (Becker et al., 2014). The binding of CA to the exchanger binding site creates a “transport metabolon” (Srere, 1987; Reithmeier, 2001; McMurtrie et al., 2004) and there appears to be variation to what extent the isoforms of CA can interact with individual members of the membrane transporter protein families. It is possible that at least a step in the regulation of vascular tone by CA is its action on one or more of these transport metabolons, although that hasn´t been demonstrated. However, it has been known for some time that CA inhibitors have an effect on bicarbonate transport across membranes in several organs (Burckhard et al., 1984; Supuran, 2016b). The catalytic activity of CA is required for maximum Cl^−^/HCO_3_^−^ transport by AE1, since inhibition of endogenous CA with acetazolamide reduces the transport activity of AE1 expressed in HEK293 cells by 50%–60% (Sterling et al., 2002). The isoform involved is CAII, and it appears that the CA isoform binds to the C-terminal tail of AE1 (Vince and Reithmeier, 2000). No interaction between the CA I isoform and membrane transporter proteins has been found (Vince et al., 2000). In addition, a functional interaction between AE1-3 chloride/bicarbonate exchangers and membrane-bound CA IV has been shown in HEK293 cells (Sterling et al., 2002), and between AE3 and CA IV, as well as the extracellular CA XIV isoform, in mouse hippocampal cells (Svichar et al., 2009). The actions of the human isoform CA II on the electrogenic Na^+^/HCO_3_^−^ co-transporter NBCe1 is somewhat controversial. Li et al. (2006) found in *Xenopus* oocytes that the current across the membrane generated by NBC activity was not affected by the presence or absence of human CA II in the cells, and that the CA inhibitor ethoxzolamide had similar effects on the co-transporter current of cells with or without human CA II. But in a subsequent similar study, although with subtle differences in methods, it was found that the membrane current in oocytes generated by NBC activity was greatly increased by injection of CA II in a dose-dependent manner, and that this increase was blocked by the CA inhibitor ethoxyzolamide (Becker and Deitmer, 2007). The differences in these findings may be due to the different levels of expression of NBCe1 in the oocytes, and different concentrations of CA II injected into the cells, but oocytes are nearly completely devoid of CA II. In addition, the two studies were performed using different holding potentials of the oocytes under voltage clamp, as well as other related differences in patch clamp parameters (Becker et al., 2014). Not only the human cytosolic CA II isoform interacts with sodium/bicarbonate co-transporters, since the membrane-associated CA IV isoform has been shown to affect NBC activity, measured as changes in pH_i_, in NBC1-transfected HEK293 cells (Alvarez et al., 2003). Another membrane-bound isoform, CA IX, has been shown to have both physical and functional interactions with NBCe1 in rat cardiac ventricular myocytes, and extracellular CA activity appears to be of great importance for NBCe1 transport function in cardiac myocytes (Orlowski et al., 2012).

The SLC26 family of Cl^−^/HCO_3_^−^ exchangers can also exchange Cl^−^/OH^−^ and in this activity the inward Cl^−^ gradient provides a driving force for a net efflux of bicarbonate via the exchangers (Mount and Romero, 2004). Some of the members of the SLC26A family show evidence of interaction with the CA II isoform, based on experiments with CA blockers like acetazolamide that reduces the exchange activity, or truncation experiments showing the CA II binding sites (Sterling et al., 2002; Alvarez et al., 2003). The mammalian Na^+^/H^+^ exchangers (NHEs) belong to the SLC9 gene family, and so far nine separate isoforms of these exchangers have been found (Slepkov et al., 2007). Their primary role is to catalyze the exchange of Na^+^ or K^+^ with H^+^ down their concentration gradients. Of the nine isoforms of NHEs only NHE1 (Slepkov et al., 2007) and NHE3 (Krishnan et al., 2015) have been shown to interact with CA. The NHE1 isoform of NHEs is ubiquitously expressed in the plasma membranes of nearly all tissue, is highly regulated by pH, and very important for regulating cell volume. Functional interactions between NHE1 and both intracellular CA II (Li et al., 2006) and extracellular CA IV (Wu et al., 1998) have been demonstrated. Physical interaction between NHE1 and CA II occurs via binding of the C-terminal region of the NHE1 to CA II (Li et al., 2006), while the binding mechanism between CA IV and NHE1 is still unknown. NHE3 has been shown to physically interact with the CA II isoform in renal proximal tubular cells at the NHE3 C-terminal, and inhibition of endogenous CA II in these cells by acetazolamide was found to decrease NHE3 activity (Krishnan et al., 2015). Thus, it is clear that nearly all of the acid/base membrane transporter families may be part of transport metabolons with carbonic anhydrases, and that CAIs can alter their activities, which raises the possibility that such metabolons may play a role in mediating the vasodilatory effects of CAIs, and by inference in regulating vascular function.

## Carbonic anhydrases in the vasculature

The enzyme carbonic anhydrase (CA) has been localized in all the main types of vascular cells and subtypes of capillaries, but in particular in the endothelial cells and smooth muscle cells. One of the earliest indications of the presence of CA outside erythrocytes in the vasculature came from its histochemical staining in amphibian and reptilian pulmonary capillaries (Fain and Rosen, 1973). Subsequent work in mammalian capillaries came from staining of rat lung and striated muscle capillaries (Ridderstråle, 1979; Lönnerholm, 1980), and the presence of the enzyme and increased expression in mammalian vascular cells with development was shown by staining of fetal arterial smooth muscle cells (Jeffery et al., 1980). Carbonic anhydrase has since been demonstrated in fenestrated juxtaepithelial capillaries of cynomolgus monkeys (Lütjen-Drecoll et al., 1985) and a variety of CA isoforms in the vascular cells of humans. The first isoform found in human capillaries was CA III (Jeffery et al., 1980), which shows a weak enzymatic activity in comparison with CA II, and subsequently the membrane-bound CA IV in the capillaries of skeletal muscle (Sender et al., 1994). The CA isoforms CA I, CA II, CA III and CA IV were found to be localized in a human umbilical vein endothelial cell line, and most probably these cells are able to synthesize these enzymes (Mathieu et al., 1995). In the cerebrovascular wall of the rat middle cerebral arteries both cytosolic isoforms CA II, CA III and CA VII, the mitochondrial isoform CA VB, and the extracellular isoforms CA IV, CA IX, CA XII and CA XIV are expressed, but the isoforms CA VII, CA XII and CA XIV appear to be absent from the rat mesenteric artery (Rasmussen and Boedtkjer, 2018). Both CA I and CA II isoforms have been found to be present in isolated bovine aortic smooth muscle cells, devoid of any erythrocytes (Berg et al., 2004). Thus, it appears that at least some of the isoforms of CA within the alpha family are present in vascular smooth muscle cells and endothelial cells. In vascular pericytes, only mitochondrial isoforms VA and VB of CA have been localized (Shah et al., 2000), and these isoforms regulate the rate of rapid oxidative metabolism of glucose in these cells. Pericytes are crucial for the survival and functionality of the blood-retinal and blood-brain barriers. Diseases including diabetic retinopathy and Alzheimer’s disease are associated with pericyte loss which is caused by hyperglycemia induced reactive oxygen species (ROS) production and oxidative stress (Hammes et al., 2002; Sengillo et al., 2013). A mitochondrial carbonic anhydrase inhibitor (mCAI), such as topiramate, can be used as an alternative to targeting coenzyme Q to inhibit the formation of oxaloacetate by preventing the production of bicarbonate. Due to the mitochondria’s impermeable membrane, bicarbonate must be produced inside of it (Price et al., 2012; Shah et al., 2013). In diabetic mice there is a loss of cerebral pericytes (Shah et al., 2013), blood-brain barrier disruption and dysfunction of neovascular units possibly due to disruption of mitochondrial CA function, since this can be prevented with selective inhibitors of mitochondrial CA isoforms like topiramate (Salameh et al., 2016; Price et al., 2017). The vascular pericytes and endothelial cells are interconnected via gap junctions at peg-socket invaginations, and via the vessel basal membrane (BM), since the pericytes are embedded in the BM and surround endothelial cells (Alarcón-Martinez et al., 2021; Dessalles et al., 2021). These interconnections are the basis for interactions between these cell types, in addition to signalling molecules released from the pericytes onto the endothelial cells (Huang, 2020; Dessales et al., 2021). Pericytes are a heterogenous group of cells with diverse functions, such as being part of blood-brain and blood-retina barriers by affecting vascular permeability and/or in regulating vascular blood flow by contraction: the expression of smooth muscle α-actin (α-SMA) and other contractile proteins such as F-actin varies greatly among pericytes (Nehls and Drenckhahn, 1991; Attwell et al., 2016; Alarcón-Martinez et al., 2021), so that some of them can contract, while others not, and thus some of them are involved in regulating capillary diameter (Alarcón-Martinez et al., 2021). It has been shown that the mitochondrial isoform CA VA regulates the rate of pericyte respiration (Patrick et al., 2015). However, the role of mitochondrial CA isoforms, including those in pericytes, in regulating vascular tone is largely unknown.

## The function of CA in vascular cells

One of the main known functions of CA in all tissue is to regulate intracellular pH and the rate of conversion between carbon dioxide (CO_2_) and bicarbonate, i.e., catalyze the reversible hydration of CO_2_ and the dehydration of HCO_3_^−^, with a two-step mechanism:

$${\text{CO}}_{2}+H_{2}O\;↔\;{\text{HCO}}_{3}^{‐}+H^{+}.$$

With these steps, CA accelerates the adjustment of the intracellular concentration of carbon dioxide and bicarbonate. The enzyme speeds up the rate of reaction in both directions by up to six orders of magnitude. Conversely, inhibition of CA or dysfunction of the enzyme slows down the mechanisms. CA can, via regular exchange of HCO_3_^−^ and H^+^ with other monovalent ions, affect transepithelial transport of these ions, and this process tends to conserve them in the cytoplasm. The involvement of CA in regulating intra- and extra-cellular pH concerns facilitating the transport function of several acid/base transporting membrane proteins, in some cases via direct binding of CA to a binding site on the transporter protein, but these processes are variable and complex (Becker et al., 2014). Furthermore, it is quite possible that some of these processes relate to the function of CA in vascular cells, directly or indirectly. The most active isoform for the carbon dioxide hydration reaction is CA IX (Hilvo et al., 2008), and it is significantly expressed in cells that are capable of rapid growth and high glycolysis as well as cancer cells that are subjected to hypoxic and acidic environments (Giatromanolaki et al., 2001). Its expression typically is associated with metastasis, cancer invasiveness, and poor disease prognosis (Ilie et al., 2010). Along these lines, CA IX protein levels rise with vascular endothelial growth factor-targeted therapy in metastatic clear cell renal cancer (mccRCC). However, CA IX protein levels are linked to a worse prognosis and resistance in many cancer cell lines (Stewart et al., 2014). This can be explained as being due to VEGF-targeted therapy which causes dynamic changes in biomarker expression, and the only tissue that can predict drug activity is tissue taken later during treatment. Thus, protein biomarkers may be identified and validated by analyzing protein expression from renal carcinoma tissue treated and untreated with VEGF, and with this approach CAIX has been found to increase with anti-VEFG-targeted therapy, as well as being an independent predictor of outcome in mccRCC (Stewart et al., 2014). It has been shown that CA IX expression is higher and declines with extracellular acidity, which is related to cell proliferation, in pulmonary microvascular endothelial cells (PMVEs) compared with pulmonary arterial endothelial cells (PAECs). Moreover, CA IX is essential for PMVECs angiogenesis during acidosis by controlling the extracellular and intracellular pH (Lee et al., 2018). CA II has been identified as an antigen which elicited humoral immune responses in melanoma patients and expressed in the endothelium of new growth vessels in cancer tissues, including melanoma, and lung, renal and esophageal cancer (Yoshiura et al., 2005). Based on a survival analysis, it has been demonstrated that endothelial CA II immunostaining is strongly related with a poor prognosis in patients with glial tumors (Haapasalo et al., 2007).

The abnormal mineral accumulation in the circulatory system is known as vascular calcification. It can occur in the heart’s valves and takes on a variety of shapes, such as medial and intimal calcification. Vascular calcification is linked to renal illness, atherosclerosis, diabetes, and several genetic disorders (Wu et al., 2013). Many human tissues, including bone and soft tissue, calcify in part due to the presence of CAs. Furthermore, this group of isoenzymes has a role in the pathological calcification of conditions such as ankylosing spondylitis, dermatomyositis, bile and kidney stone development, and calcification associated with cancer (Adeva-Andany et al., 2015; Zamanova et al., 2019). CA I catalyzes the hydration of CO_2_ reversibly to produce HCO^3-^ which quickly binds to calcium ions to form calcium carbonate (Supuran, 2008b). Calcium carbonate in turn is a base for calcium phosphate (hydroxyapatite) deposition, which in human atherosclerotic plaques has been found to amount to 71% of their chemical composition, while calcium carbonate amounts to about 9% (Adeva-Andany et al., 2015). But CA may contribute to vascular calcification indirectly, by supplying CO_2_ to g-glutamyl carboxylase, whose enzymatic action activates several proteins that may be involved in calcification, but less is known about the involvement of CA in that process (Adeva-Andany et al., 2015). In a clinical study, overexpression of CA I has been found in the synovium of ankylosing spondylitis patients. This finding suggests that CA I may accelerate calcium production and bone resorption, which are two crucial processes in bone formation (Chang et al., 2010). In addition, it has been found that CA I protein levels were increased in tissues and blood samples from patients with breast cancer, suggesting that CA I may play a crucial role in migration, cell death, and calcification of cancer cells by controlling the expression of X-box binding protein 1 (*XBP1*) and androgen receptor (*AR*) (Zheng et al., 2015). It has been demonstrated that CA I protein levels and CA I-mediated calcification are significantly correlated with atherosclerosis development in a mouse model of atherosclerosis (AS). In the same study, methazolamide, a CAI that significantly reduced aqueous fluid secretion and intraocular pressure in the eye, dramatically reduced AS, inhibited CA I protein levels, and reduced the release of proinflammatory cytokines (Yuan et al., 2019), indicating that methazolamide might be an attractive therapeutical agent to prevent this condition.

Regarding the regression or decalcification of minerals that are ectopically precipitated, not much is known. During bone remodeling, osteoclasts disintegrate minerals by generating an acidic environment in the resorption lacuna. In this mechanism, the synthesis of H^+^ and the movement of protons are crucially dependent on CA II and vacuolar H^+^-ATPase, respectively (Lehenkari et al., 1998; Maxson and Grinstein, 2014). CA II is crucial for osteoclast development and bone resorption. CA II-deficient mice show histopathological changes including a visible age-dependent calcification of small arteries in several organs (Spicer et al., 1989). It has been reported that macrophages show high CA II expression and successfully demineralize the ectopic calcification. Thus, CA II may be a potent therapeutic target for the treatment of vascular calcification (Barinda et al., 2017). Recently, it has been demonstrated that M1-phenotype macrophages enhance the production of TNF-α, a proinflammatory cytokine, to promote vascular calcification in human vascular smooth muscle cells (VSMCs) by upregulating their CA I and CA II expression (Song et al., 2023). Thus, it is clear that the function of CA in vascular cells is complex, involving a variety of processes, but it may be that one additional function of the enzyme in these cells is to contribute to the regulation of vascular function and blood flow.

## Carbonic anhydrase and blood flow

It has been known for some time now that CA plays a role in regulating blood flow, and that the enzyme located in vascular cells is a key contributor in that process (Rassam et al., 1993; Grossman and Koeberle, 2000; Stefánsson et al., 2005). In most of the organs of the body that have been examined, inhibition of CA has been found to affect vessel diameter and blood flow, indicating that the enzyme may be involved either directly or indirectly in regulating vascular function. However, differences have been found between organs when it comes to the effects of CA inhibition on vascular function and blood flow. It has been well known for some time that CA activity (tested by CA infusion or that of CA inhibitors) varies greatly between organs, and species (Crandall and O’Brasky, 1978; O’Brasky et al., 1979; O’Brasky and Crandall, 1980). In the rat, the CA inhibitor acetazolamide induces a dose-dependent increase in cerebral blood flow, and in the regional blood flow of the kidneys and liver but has no effect on blood flow in the stomach wall and abdominal muscle (Taki et al., 2001). Similarly, acetazolamide has no significant effect on pancreatic or duodenal blood flow in normal rats (Carlsson et al., 1998). Still, CA isoforms CA I and CA II have been localized in the human gastrointestinal tract, including stomach and colon with high CA activity, but only CA I in superficial capillaries of all regions of the tract (Lönnerholm et al., 1985). It may be that the apparent absence of other isoforms of CA than CA I in capillaries of the gastrointestinal tract is the reason why blood flow is not affected by CA inhibition there. It is well-established that CA inhibitors have strong effects on respiratory functions and pulmonary blood flow, and drugs like acetazolamide and benzolamide are used as treatment for high altitude acclimatization and acute mountain sickness (Swenson, 2014). One consequence of hypoxia that occurs when moving from sea level to higher altitudes is hypoxic pulmonary vasoconstriction (HPV) (Teppema et al., 2007; Swenson, 2013). The stimulus for HPV is most likely reduced oxygen levels, since under normobaric hypoxia similar effects on the pulmonary vasculature, i.e., increased pulmonary vascular resistance (PVR) and pulmonary artery pressure (PAP) as well as pulmonary vasoconstriction occur in mammals (Swenson, 2013). HPV can be reduced by systemic acetazolamide and methazolamide in volunteers (Teppema et al., 2007; Boulet et al., 2018), and the pulmonary vasodilation during hypoxia can be achieved by inhalation of acetazolamide in dogs, although it still causes systemic CA inhibition (Pickerodt et al., 2014). It is thus clear that CA inhibitors affect pulmonary vascular function and blood flow by inducing vasodilation.

The vast majority of studies that have been done on the effects of CA inhibitors on blood flow have been done in the brain and the eye. In the brain, the cerebral capillaries are well known to be influenced by CA activity, and CA inhibitors such as acetazolamide induce elevation of human cerebral blood flow (Vorstrup et al., 1984; Okazawa et al., 2001), and in a dose-dependent manner (Grossman and Koeberle, 2000). Indeed, acetazolamide IV infusion has been used as a clinical test in neurology to assess *cerebrovascular reserve*, i.e., to what extent cerebral perfusion can increase from a baseline value with stimulation by either acetazolamide infusion or CO_2_ inhalation (Settakis et al., 2003). In addition, the putative use of CAIs for management of cerebral ischemia has been suggested, based on the cerebrovascular dilatory effect of these compounds, even in induced middle cerebral artery occlusion in rats (Di Cesare Mannelli et al., 2016; Bulli et al., 2021). In the eye the role of CA in retinal vascular function has been studied extensively (Grunwald and Zinn, 1992; Rassam et al., 1993; Stefánsson et al., 2005; Pedersen et al., 2005). It is clear that CA inhibition induces increased blood flow in the retinal vasculature (Rassam et al., 1993; Pedersen et al., 2005; Siesky et al., 2009), increases oxygenation over the optic nerve and retina (Stefánsson et al., 1999; Stefánsson et al., 2005) and these effects are most likely due to direct vasodilatory effects of these compounds on retinal vessel walls (Josefsson et al., 2004; Torring et al., 2009; Eysteinsson et al., 2022). But in the eye, differences have been found in the effect of CAIs on the different ocular vascular beds, and between studies; some have found an increase in choroidal blood flow induced by CAIs (Dallinger et al., 1998), while others have found no effect (Haustein et al., 2013) or a reduction in choroidal blood flow with CA inhibition (Zinkernagel and Ebneter, 2009). Measuring choroidal blood flow is technically more difficult than retinal blood flow and may partly explain the conflicting results. The effects of CA inhibitors on the retinal vasculature have been examined extensively and therefore requires discussion here in greater detail.

## Carbonic anhydrase and the retinal vasculature

The human eye contains at least four isoforms (CA I, II, IV, and XIV). The main CAs isoforms expressed in the eye with their subcellular and cellular localization and function are shown in Table 1.

**TABLE 1:** CAs isoforms expressed in the human eye and their characteristics.

CAs isoforms	Subcellular localization	Cellular localization in the eye	Function in the eye	Source
I	Cytosolic	Corneal endothelial cells, lenticular cells, capillary endothelial cells, and choroidal cells	Vasoconstriction and vasodilation	Wistrand et al. (1986), Berg et al. (2004)
II	Cytosolic	Ciliary epithelial cells, Müller cells, and photoreceptor cells	Aqueous humor production, controlling intraocular pressure, fluid flow and acid-base balance regulation	Wistrand et al. (1986), Wu et al. (1998), Kaur et al. (2002)
IV	Extracellular membrane-anchored	Lens epithelial cells, fiber cells and choriocapillaries	Fluid flow and acid-base balance regulation	Hageman et al. (1991), Wu et al. (1998)
XIV	Extracellular membrane-bound	RPE, Müller cells, and astrocytes	Increasing subretinal fluid absorption, improve blood flow, facilitate CO_2_ removal from the retina and regulate photoreceptor function	Nagelhus et al. (2005)

The low-activity form of cytosolic isoenzyme CA I, which is found in vascular smooth muscle cells and vascular endothelial cells as well as in red blood cells and digestive tract cells, is responsible for controlling both vasoconstriction and vasodilation (Berg et al., 2004). CA I has been found to be expressed in the following type cells of the eye: the corneal endothelial, lenticular, capillary endothelial cells, and choroidal cells (Wistrand et al., 1986). In many different types of organs and cells, CA II is a highly active form that is generally present, particularly in erythrocytes where it promotes CO_2_ transport (Wistrand, 1981). In the retina, CA II has found to be expressed in ciliary epithelial cells, Müller cells, and a subset of cone photoreceptor cells (Wistrand et al., 1986). Aqueous humor production and intraocular pressure are regulated by CA II. Acetazolamide, methazolamide, and dichlorphenamide are CA II inhibitors that have been demonstrated to show excellent efficiency in treating glaucoma, a condition characterized by an excess of aqueous fluid and increased intraocular pressure (IOP) (Kaur et al., 2002). CA IV is an isoenzyme with high activity that is extensively distributed. CA IV is the first membrane-associated isoenzyme discovered in the human eye, residing in the lens’ epithelial and fiber cells as well as the choriocapillaris (Hageman et al., 1991). It has been proposed that CA IV, in addition to CA II, has a considerable impact on fluid flow and acid-base balance (Wu et al., 1998). CA XIV, an extracellular membrane-bound CA, has been shown to be abundant in the retinal pigment epithelium (RPE), Müller cells, and astrocytes. This suggests that targeting CA XIV may increase subretinal fluid absorption and improve blood flow (Nagelhus et al., 2005).

It has been well established that CA is present in the retinal vasculature (Berg et al., 2004), and that inhibition of the enzyme induces a vasodilation, increased oxygen tension in the retina and optic nerve (Stefánsson et al., 1999) and enhanced blood flow in retinal vessels (Stefánsson et al., 2005). The retinal vasculature supplies oxygen and nutrients to the neuroretina and is structured to meet the extensive metabolic demands of that neural tissue, while having relatively sparse distribution of vessels, ensuring minimum optical interference to the light that traverses through the retina to the photoreceptors. The microcirculation of the retina derives from the central retinal artery in humans, and branches into four large intraretinal arteries, each of which serves one quadrant of the retina. These arteries then branch into smaller arterioles that feed into the retinal capillary beds. The retinal arteries and arterioles travel in the ganglion cell layer. The retinal circulation is a true end artery system, with all the blood from the retinal arteries passing via the capillary beds to retinal venules and the central retinal vein. With fundus imaging, the retinal circulation can be easily viewed and examined, and structural and functional parameters such as vessel diameter (Pedersen et al., 2005; Daníelsson et al., 2022; García-Llorca et al., 2023) and alterations in blood flow (Marino et al., 2021) can be readily measured. The retinal vasculature tends to maintain a steady level of blood flow and oxygenation in the retina, by intrinsic autoregulation (Riva et al., 1981; Dumskyj et al., 1996) in response to changes in perfusion pressure, intraocular pressure, or blood gasses (la Cour et al., 2000), but the myogenic response, considered to be the basis of autoregulation, is held to be intrinsic to vascular smooth muscle cells (Davis and Hill, 1999).

Topical CAIs have also been widely shown to be capable of raising indicators of ocular blood flow via chemically mediated vasodilatory pathways, although their specific relevance to the beginning and course of open angle-glaucoma (OAG) is still unknown (Stoner et al., 2022). The investigation of more current topical CAI formulations incorporating nitric oxide moieties or novel delivery techniques (Lusthaus and Goldberg, 2017; Schehlein and Robin, 2019) may result in a better usage of CAIs by clinicians in the future (Stoner et al., 2022). Several studies have demonstrated that topical or systemic administration of CAIs increases retinal blood flow (Barnes et al., 2000; Iester et al., 2004). Moreover, topical CAIs accelerate the retinal circulation, central retinal, and short posterior ciliary arteries rates of ocular blood flow (Siesky et al., 2009). The normal retina’s adhesion to the RPE is maintained by a number of interrelated mechanisms (Zauberman, 1979). Acetazolamide can positively affect retinal adhesion and subretinal fluid resorption in rabbits (Marmor and Maack, 1982; Wolfensberger et al., 2000). As a result, CAIs may play a role in increasing adhesiveness by accelerating RPE transport. CAIs in clinical use such as dorzolamide dilate the central retinal capillaries and the time course of this dilation resembles the rise in retinal oxygen tension. Thus, CA inhibitor-induced oxygen tension elevation can be explained by an enhanced oxygen supply to the retina (Pedersen et al., 2005). It was proposed at first that perhaps CO_2_ buildup, a recognized vasodilatory stimulus, might be the cause of CA inhibitor-induced vascular dilatation (Pedersen et al., 2005). In addition, CAIs are capable of lowering the pH in the extracellular fluid, while increasing in the intracellular fluid. Such pH changes have been shown to coincide with an increase in capillary diameter in rat retinal whole mounts (Reber et al., 2003). Thus, CAIs may relax pericytes and possibly enhance retinal blood flow, although the exact vascular role of CA isoforms in pericytes is still unclear. Dorzolamide, however, has also been demonstrated to dilate isolated pre-contracted retinal arteries, even with the pH and CO_2_ kept at steady levels in the extracellular fluid around the vessels (Josefsson et al., 2004; Torring et al., 2009). Therefore another mechanism involved could be a direct vasodilatory action unrelated to variations in extracellular pH and CO_2_ (Josefsson et al., 2004; Torring et al., 2009; Eysteinsson et al., 2022; 2023). However, the mechanisms are not yet fully understood, although several attempts have been made to clarify the issue.

## Putative mechanisms of the action of CA on vascular tone

The mechanism behind the well-established direct CA action on vascular tone (Josefsson et al., 2004) is still unknown. Several hypotheses have been put forward, and some of them tested with inconclusive results. One of the first hypotheses tested related to the basic actions of the enzyme, in catalyzing the conversion of CO_2_ and HCO_3_^−^, which leads to acidification (Reber et al., 2003). Are changes in intracellular and/or extracellular pH the basis of the role of CA in regulating vascular tone? The answer is that alterations in extracellular pH induced by CA or inhibition of CA is not involved in regulation of vascular tone, since even if the pH is maintained at 7.4 in the extracellular fluid by HEPES or similar buffers, CA inhibitors still induce vasodilation in pre-contracted arterial segments (Josefsson et al., 2004; Torring et al., 2009). When CA inhibitors induce vasodilation in retinal arteries there is an increase in the intracellular acidification of smooth muscle cells in the vessel walls (Reber et al., 2003), but there is no direct relation between vascular relaxation and intracellular acidosis in these vessels (El-Galaly et al., 2014). Changes in CO_2_ concentration due to CA action does not play a role in regulating vascular tone, since CA inhibitors still induce vasodilation under normocapnia and hypercapnia, and with nominal absence of CO_2_ and HCO_3_^−^ (Torring et al., 2009). The fact that most aspects of the main action of CA as an enzyme, i.e., regulation of intracellular pH, and CO_2_ and HCO_3_^−^ concentration, do not appear to be involved in control of vascular tone has led to the hypothesis that the vasodilating effect of CA inhibitors involves other mechanisms than inhibition of the enzyme (Torring et al., 2009). What mechanisms might then be involved is, however, unclear. Adjacent tissue to the vessels and pericytes may interact with the vascular walls and thus affect vascular tone, and in the retina and brain there is neurovascular coupling that plays a major role in regulation of vascular tone and blood flow (Attwell et al., 2010; Kur et al., 2012). It was hypothesized that CAIs exert their vasodilatory effect via neurovascular coupling, but when neural tissue is removed from vessels isolated in a tissue bath the vasodilatory effects of CAIs are reduced, but not completely removed (Kehler et al., 2007). This indicates that the regulatory function of CA on vascular tone is in part independent of neurovascular coupling, and that processes on the vessel wall are implicated. The vessel wall includes smooth muscle cells, pericytes and the vascular endothelial cells, and CA may regulate vascular tone through mechanisms in some or all these cell types.

The endothelium affects vascular tone and blood flow by the release of nitric oxide (NO) onto pericytes and vascular smooth muscle cells, inducing relaxation (Palmer et al., 1987; Zhao et al., 2015). NO is released from the vascular endothelium (Palmer et al., 1987), and it has several effects on vascular cells, including affecting vascular tone by inducing relaxation of vascular smooth muscle cells by activating soluble guanylyl cyclase, which in turn leads to increase in the synthesis of cGMP, eventually leading to vasorelaxation (Bolotina et al., 1994). The release of NO from the vascular endothelium may be affected by the actions of CA, such that CA inhibition increases the release of NO from the endothelium. NO has also been found to inhibit voltage-gated Ca^2+^ channels and thus decrease cytosolic Ca^2+^ concentration, and to activate Ca^2+^-dependent potassium channels on vascular smooth muscle cells (Bolotina et al., 1994; Zhao et al., 2015). Both these effects on ion channels lead to vasorelaxation. However, it is likely that activation of guanylyl cyclase by NO is the mechanism involved, since inhibition of guanylyl cyclase reduces the relaxing effect of CAIs to the same degree as inhibition of NO synthase (Kringelholt et al., 2012; El-Galaly et al., 2014). However, the exact link between vascular CA activity and NO release (and NOS activity) is still unknown. Earlier studies indicated that blockage of NO synthase (NOS) and thus reduction in NO synthesis does not block or reduce the 62% increase in cerebral blood flow (CBF) induced by the CAI acetazolamide in rats (Wang et al., 1992), although a later study in Wistar rats, using laser Doppler to measure rCBF found that L-NNA, an inhibitor of all NOS, prevented stimulation of CBF by acetazolamide, suggesting that NO may act as modulator of the acetazolamide induced rCBF response (Tuettenberg et al., 2001). Similar studies in humans indicated that the acetazolamide-induced increase in CBF and ocular blood flow is independent of NO (Kiss et al., 1999), but it may be that the NOS inhibitor used in that study, L-NMMA, did not block all the NO synthetases as does L-NNA. It should be noted, however, that evidence shows that resistance vessels in humans are in fact constantly dilated to a degree by regular release of NO (Vallance et al., 1989; Schmetterer and Polak, 2001). But there may be variations between vascular structures to what extent if any CA affects vascular tone via endothelial NO release. The endothelium clearly plays a role, since inhibition of the formation of nitric oxide (NO) by L-NAME, a NO synthase inhibitor, or blockage of the NO receptor guanylyl cyclase by ODC reduces the vasorelaxation induced by CAIs but does not fully block that effect in isolated retinal arteries (El-Galaly et al., 2014). However, L-NAME fully blocks the vasorelaxant effect of CAIs on intraocular porcine ciliary arteries (Kringelholt et al., 2012), once again raising the question of whether the NO synthase blockers used in such studies are selective or block all the synthetases. It also raises the question of whether the NOS involved may vary between organs and vessel types. It has been suggested that endothelial NO is generated by the actions of carbonic anhydrase from nitrite, and that this metabolic action relates to the vasodilation induced by CAIs (Aamand et al., 2009), but this suggestion has been challenged (Andring et al., 2018; Wang et al., 2020).

One possible mechanism through which CA may act on vascular tone is via modulation of the functions of ion channels on vascular cells. It has been shown that the CAI benzolamide blocks voltage-gated Ca^2+^ channels expressed on HEK293 cells (McNaughton et al., 2004) and on isolated hippocampal neurons (Gottfried and Chesler, 1995), but it is not known if such CAI actions mediate their relaxation of vascular tissue. The SK_Ca_ channel blocker charybdotoxin has been found to partially block vasorelaxation induced by CAIs on isolated mesenteric arteries (Pickkers et al., 2001), and the BKCa channel blocker iberiotoxin has a similar effect on CAI induced vasorelaxation of rat aorta (Carre et al., 2015). We find, in isolated segments of porcine retinal arteries by means of small wire myography, that neither charybdotoxin or iberiotoxin blocks or in any way affects the vasodilatory effects of CAIs, and that none of the blockers or activators of potassium channels tested so far has any effects on CAI action on vascular tone in arterial segments (Eysteinsson et al., unpublished data). Thus, it is unclear if CA acts on vascular function by altering or modifying the activity of specific ion channels. Despite the fact that the interplay between CA and a variety of membrane transporters, or transport metabolons, is well established (Becker et al., 2014), as described above, the role of this interplay in regulating vascular tone and function is largely unknown (but see Rasmussen and Boedtkjer, 2018).

The role of individual CA isoforms in regulating vascular function and tone is largely unknown. However, the vasoactive actions of CAIs with different binding affinity for separate isoforms, cytosolic or membrane bound, with enhanced lipophilicity or other distinct qualities may provide important indications (Eysteinsson et al., 2019; 2022). Measured by small vessel myography from vessel segments the potency of CAIs varies greatly, and EC_50_ for vasodilation of pre-contracted vessels by some inhibitors such as those depicted in Figure 1A can be as low as 10^−5^ M, depending on their structure (Eysteinsson et al., 2019). These compounds include the hydrophilic benzolamide **1** (a derivative of acetazolamide, but more acidic than the parent drug), dorzolamide **2**, another hydrophilic, second generation CAI (Supuran, 2008a), as well as the highly lipophilic di-propargyl derivative of benzenesulfonamide **3**. Furthermore, coumarins inciorporating the propargyl moiety (as ether) in various positions of the heterocyclic ring, of types **4–6**, were also included in the first study (Eysteinsson et al., 2019). However, although lipophilicity (measured as LogP) plays a role in determining the EC_50_ for vasodilation by CAIs, when tested by compounds such as the ones depicted in Figure 1B that have a modified structures to enhance their lipophilicity, it has been observed that lipophilicity not the sole determining factor (Eysteinsson et al., 2022). Indeed derivatives **7–14** (Figure 1B) are all benznesulfonamides which incorporate both hydrophilic (thioureido, seleno-etheracylated selenoureido moieties), as well as highly lipophilic tails, such as those present in **12–14** of the dimethoxybenzamido-phenylselenoether, *n*-penthyl-thioether or phenethylthioether types. It seems however that extracellular CA isoforms, or binding sites on membrane bound isoforms outside the cytosol are unlikely to play a role in regulation of vascular tone function, since only membrane permeable CAIs elicit the vasodilation. Indeed, membrane impermeable CAIs like the compounds depicted in Figure 1C of types **15–17**, which are again benzenesulfonamides, but possessing positively charged moieties at the physiological pH due to their biguanidyl functionalities, are not able to induce vasodilation in isolated pre-contracted porcine retinal arterial segments (Eysteinsson et al., 2023).

## Conclusion

In this review we have examined the importance of CA in regulating vascular tone and blood flow in the vasculature, the effects of CA inhibitors on vascular function, and through what mechanisms the enzyme may regulate vascular function. It is well established that CA inhibitors affect vascular function by inducing vasodilation in both arteries and veins in many organs of the body, and it is likely that this action is due to direct inhibition of the isoforms expressed in the vessel walls of these vessels. But still, several issues are unresolved, including why CAIs do not induce vasodilation and altered blood flow in all organs of the body, only some. The physiological mechanism by which the enzyme and its inhibitors exert their effect on vascular tone is still unknown. Several hypotheses have been proposed and tested, some of which are related to the basic functions of CA, like regulation of intracellular and extracellular pH and levels of CO_2_ and bicarbonate, but it appears that the actions of CA and inhibitors on vascular tone are independent of these functions. It is likely that cytosolic isoforms of CA are primarily involved, but it is still not clear which of them are most important. There are indications that in some cases CA inhibitors exert their effect on vascular tone via actions on calcium activated potassium channels, but it is unlikely to be the only mechanism involved. The search for other ion channels that may be involved has so far been unsuccessful. One of the functions of carbonic anhydrase is to affect the activity of ion exchangers, via transport metabolons, but so far little or no work has been done to address the possibility that the vasodilatory effects of some CAIs may be mediated through their actions on ion transport, and that CA may regulate vascular function via actions on transport metabolons. We still do not know which isoforms of CA are critically important for vascular function and regulation of vascular tone, although it is likely that cytosolic isoforms, or intracellular binding sites of membrane bound isoforms are involved. Thus, there are several hypotheses on the mechanisms that could be put forward and tested in the future before we have a clear picture of how CA and its inhibitors affect vascular function.
